# Toward a digital child health booklet

**DOI:** 10.1093/eurpub/ckac129.602

**Published:** 2022-10-25

**Authors:** J Dratva, S Stronski

**Affiliations:** 1 Institute of Public Health, ZHAW Zurich University of Applied Sciences, Winterthur, Switzerland; 2 Medical Faculty, University of Basel, Basel, Switzerland; 3 “pediatrie schweiz”, Swiss Paediatric Society, Bern, Switzerland; 4 School Health Services, Health Services City of Bern, Bern, Switzerland

## Abstract

Public health, paediatrics as well as other health professionals share the interest in providing services and improving conditions to ensure life-long health and well-being of children and adolescents. In this interprofessional setting and aim, parents are central partners, as are adolescents when they take over the responsibility of their own health. Ensuring the availability of health data for parents and adolescents at any given time and place is a key factor to empower and improve health management and literacy, providing continuity of health information along the care chain, and analysing health data of healthy and sick children is of high importance. The digital booklet will have a positive impact on sharing of health information among care professionals, thus ensuring continuity of care and limiting redundancy of investigation, and in addition provide data for public health research and monitoring of health and determinants. The Swiss Society of Paediatrics, the ZHAW/Institute of Public Health and the Kollegium of Hausarztmedizin (general practices) founded an association to digitalize the current paper child and adolescent health booklet with the aims:

1. Empower parents as ‘owners’ of health data to take responsibility and have greater autonomy in managing their child's health and illness

2. Provide a digital infrastructure for

– low-threshold and reliable source of advice

– easy update of data/information, digital communication with parents

– sharing of data with professionals and non-professionals involved in care of child.

3. Monitoring of children's health data (parental consent provided)

The speakers with present their collaboration and project, its current status, as well challenges and solutions found.

